# Modulatory effect of MG-132 proteasomal inhibition on boar sperm motility during *in vitro* capacitation

**DOI:** 10.3389/fvets.2023.1116891

**Published:** 2023-03-23

**Authors:** Lenka Hackerova, Barbora Klusackova, Michal Zigo, Natalie Zelenkova, Katerina Havlikova, Romana Krejcirova, Marketa Sedmikova, Peter Sutovsky, Katerina Komrskova, Pavla Postlerova, Ondrej Simonik

**Affiliations:** ^1^Department of Veterinary Sciences, Faculty of Agrobiology, Food, and Natural Resources, Czech University of Life Sciences Prague, Prague, Czechia; ^2^Division of Animal Sciences, University of Missouri, Columbia, MO, United States; ^3^Department of Obstetrics, Gynecology and Women's Health, University of Missouri, Columbia, MO, United States; ^4^Laboratory of Reproductive Biology, Institute of Biotechnology of the Czech Academy of Sciences, BIOCEV, Vestec, Czechia; ^5^Department of Zoology, Faculty of Science, Charles University, Prague, Czechia

**Keywords:** ubiquitin-proteasome system, hyperactivation, phosphorylation, reproduction, sperm physiology, cluster analysis

## Abstract

A series of biochemical and biophysical changes during sperm capacitation initiates various signaling pathways related to protein phosphorylation leading to sperm hyperactivation, simultaneously with the regulation of proteasomal activity responsible for protein degradation and turnover. Our study aimed to unveil the role of the proteasome in the regulation of boar sperm motility, hyperactivated status, tyrosine phosphorylation, and total protein ubiquitination. The proteolytic activity of the 20S proteasomal core was inhibited by MG-132 in concentrations of 10, 25, 50, and 100 μM; and monitored parameters were analyzed every hour during 3 h of *in vitro* capacitation (IVC). Sperm motility and kinematic parameters were analyzed by Computer Assisted Sperm Analysis (CASA) during IVC, showing a significant, negative, dose-dependent effect of MG-132 on total and progressive sperm motility (TMOT, PMOT, respectively). Furthermore, proteasomal inhibition by 50 and 100 μM MG-132 had a negative impact on velocity-based kinematic sperm parameters (VSL, VAP, and VCL). Parameters related to the progressivity of sperm movement (LIN, STR) and ALH were the most affected by the highest inhibitor concentration (100 μM). Cluster analysis revealed that the strongest proteasome-inhibiting treatment had a significant effect (*p* ≤ 0.05) on the hyperactivated sperm subpopulation. The flow cytometric viability results proved that reduced TMOT and PMOT were not caused by disruption of the integrity of the plasma membrane. Neither the protein tyrosine phosphorylation profile changes nor the accumulation of protein ubiquitination was observed during the course of capacitation under proteasome inhibition. In conclusion, inhibition of the proteasome reduced the ability of spermatozoa to undergo hyperactivation; however, there was no significant effect on the level of protein tyrosine phosphorylation and accumulation of ubiquitinated proteins. These effects might be due to the presence of compensatory mechanisms or the alteration of various ubiquitin-proteasome system-regulated pathways.

## 1. Introduction

Mammalian spermatozoa are unable to fertilize an oocyte immediately after ejaculation, even though they acquire the potential for progressive motility and *in vitro* fertilizing ability once they reach the distal epididymis (1–3). Chang (4) and Austin (5) showed that spermatozoa acquire fertilizing ability *in vivo* only after staying in the female oviduct, where they undergo a series of physiological, structural, and molecular changes collectively called sperm capacitation. At the molecular level, the capacitation process is associated with the cholesterol efflux from the sperm plasma membrane occurring after the removal of decapacitation factors (6), such as proteins/glycoproteins of seminal plasma adsorbed on the surface of freshly ejaculated spermatozoa responsible for the membrane stabilization and formation of the oviductal sperm reservoir (7). Removal of decapacitation factors from the sperm surface may also be regulated by the ubiquitin-proteasome system (UPS) (8, 9). The 26S proteasome, a multi-subunit holoenzyme is an essential component of the ubiquitin and ATP-dependent proteolytic pathway, which is responsible for the regulated substrate-specific proteolysis of most cellular proteins (8, 10). Proteasomes are present in the nucleus and cytoplasm of all eukaryotic cells and their presence has also been demonstrated in spermatozoa (11–13). It is a highly organized multiprotein-multicatalytic protease complex, which comprises a 20 S catalytic core capped on one or both sides by a 19S regulatory particle that modulates its activity (8, 14–16). A protein that is destined for degradation by UPS is recognized and distinguished from other proteins *via* polyubiquitination, a covalent post-translational modification that is considered one of the key factors in the activity and stability of proteins (17).

During sperm capacitation, UPS can directly or indirectly modulate protein serine and threonine phosphorylation by degrading serine phosphatases, threonine kinases, and proteins phosphorylated on threonine residues (18). Nevertheless, the 26S proteasome has been reported to be phosphorylated by protein kinase A (PRKA) during sperm capacitation, leading to the increased chymotrypsin-like activity of the 20S proteasomal core (19, 20). This finding has been confirmed by the phosphorylation of several proteasomal core subunits at Tyr and Ser / Thr residues (18). Tyrosine phosphorylation (pY) of sperm flagellar proteins plays an important role in sperm hyperactivation (21) leading to the release of the spermatozoa from the oviductal reservoir during capacitation and facilitating sperm penetration through the cumulus oophorus and zona pellucida of an oocyte (7, 22). Hyperactivated mammalian sperm movement is defined as a less progressive, high-energy movement with asymmetric flagellar oscillation (23–25). Mortimer (26) states that hyperactivated motility occurs when the flagellum is beating with high amplitude in the proximal region. However, the exact pattern of movement of hyperactivated spermatozoa is difficult to define, because it varies between species as well as in the density and length of the flagellum and the physical environment in which the male gametes are currently moving (24). The movement trajectories are complex and there is no single kinematic parameter that would reliably define hyperactivated motility. Therefore, it is necessary to analyze selected sperm kinematic parameters with a comprehensive approach and to use logical arguments during the final evaluation of their biological meaning (27). Sperm motility is crucial for the final successful delivery of the paternal DNA to the oocyte at the fertilization site. On their journey, spermatozoa are exposed to varied direct and indirect influences which favor the selection of the fittest spermatozoa for oocyte fertilization. To our knowledge, there is only one study at the submission of this manuscript specifically exploring the effect of proteasome inhibition on sperm motility (28).

The UPS activity during sperm capacitation may be modulated by inhibitors of enzymes involved in the tagging of protein substrates with ubiquitin (ubiquitin-activating, conjugating, and ligating enzymes) or directly affected at the level of protein hydrolysis within the 20S core (8, 29). Those results are consistent with the study of Morales et al. (30), where the proteasomal inhibitor epoxomicin significantly reduced protein phosphorylation on serine residues during human sperm capacitation. These results suggest that, although the proteasome is a substrate for phosphorylation, it can directly or indirectly control its protein kinase interactors through a feedback loop (30). Proteasomal inhibitor MG-132 is the validated, commercially available peptide aldehyde, which inhibits chymotrypsin-like activity and caspase-like activities of the 20S core and is therefore widely used to study proteasome involvement in various aspects of cellular processes (31). Even though the proteasome has multiple active sites, inhibition of all of them is not necessary to significantly reduce protein degradation. This is evidenced by the fact that inhibition of chymotrypsin-like sites/subunits or their inactivation by mutations resulted in a significant reduction in the rate of protein degradation (32, 33). Our comprehension of UPS activity in sperm capacitation is far from complete. Thus, the aim of this study was to assess motility patterns in proteasomally-inhibited spermatozoa during sperm capacitation and to evaluate the capacitation-associated processes such as protein tyrosine phosphorylation and protein ubiquitination.

## 2. Material and methods

Whole ejaculates from fertile Duroc boars (number of boars, *n* = 8; four boars with 2 collections and four boars with 3 collections) used for commercial artificial insemination, were provided by Insemination station Skrsin (LIPRA PORK a.s., Rovensko pod Troskami, Czech Republic) from December 2020 to July 2022. The semen was transported in a styrofoam box at a constant temperature. Unless otherwise stated, all chemicals used in this study were purchased from Sigma-Aldrich (MO, USA). The sperm concentration and motility of ejaculates were evaluated by conventional andrological methods with the use of a light microscope. Spermatozoa were separated from seminal plasma by centrifugation at 300 × g. Spermatozoa were washed three times with the non-capacitating medium (NCM, HEPES-buffered Tyrode Lactate solution supplemented with polyvinyl alcohol void of calcium and bicarbonate; 114 mM NaCl, 3.2 mM KCl, 0.34 mM NaH_2_PO_4_, 10 mM Na-lactate, 10 mM HEPES, 12 mM Sorbitol, 21 mM Gentamicin, 0.174 mM Penicillin, 0.01% (w/v) polyvinyl alcohol, 0.5 mM MgCl_2_, 0.2 mM Na-pyruvate; pH 7.4) at 300 × g, at room temperature, and adjusted to the final concentration of 5 × 10^7^ sperm/mL. After washing, the sperm concentration was estimated again under a light microscope using a Bürker chamber.

### 2.1. Sperm *in vitro* capacitation (IVC)

Washed spermatozoa were capacitated in TALP-based sperm capacitation medium (NCM supplemented with 5 mM Na-pyruvate, 2% (w/v) bovine serum albumin, 2 mM CaCl_2_, 2 mM sodium bicarbonate, and 11 mM D-glucose). Sperm samples were divided into six experimental groups: (i) spermatozoa with capacitation medium (CM) without proteasomal inhibitor MG-132 and dimethyl sulfoxide (DMSO); (ii) spermatozoa in CM with 1% (v/v) DMSO; (iii–vi) following experimental groups of spermatozoa in CM with various concentrations of MG-132 inhibitor and DMSO: (iii) 10 μM and 0.1% DMSO (MG10); (iv) 25 μM and 0.25% DMSO (MG25); (v) 50 μM and 0.5% DMSO (MG50); (vi) 100 μM and 1% DMSO (MG100). All sperm treatment groups underwent capacitation for 3 h at 38 °C with 5% (v/v) CO_2_. At hourly intervals, motility measurements were performed by the CASA system. Samples were processed for Western blot (WB) detection of protein tyrosine phosphorylation and ubiquitination, and image-based flow cytometry (IBFC) measurement of sperm viability and surface ubiquitination. For time 0 h, spermatozoa were evaluated after 10 min of incubation with the MG-132 inhibitor, DMSO, or CM.

### 2.2. Motility assessment

Sperm motility and individual kinematic parameters were evaluated using the Computer Assisted Sperm Analysis (CASA) system module as part NIS Elements Ar 4.50. developed by Laboratory Imaging Ltd. (Prague, Czech Republic; https://www.nis-elements.cz/en/niselements/nis_advanced_research), and a phase contrast microscope (Nikon Eclipse E600, Japan) with a heating plate, a negative phase contrast objective, and a digital camera with 50 FPS (DMK 23UM021 Imaging Source, Germany). A 5 μl sperm sample was placed in a preheated (38°C) Leja counting chamber (Leja, Netherlands). Subsequently, in 6 random fields, motility was recorded and at least 200 cells per field were analyzed. Six resulting kinematic parameters, namely linearity (LIN), straightness (STR), the amplitude of lateral head displacement (ALH), straight-line velocity (VSL), average path velocity (VAP), and curvilinear line velocity (VCL), were subsequently subjected to statistical analysis. Spermatozoa with a threshold value of VAP ≥ 15 μm and STR 80% were considered progressively motile. Spermatozoa during IVC were evaluated (see sperm *in vitro* capacitation) at time zero and then every hour for all samples separately. Inhibitor reversibility was evaluated after sperm IVC (2 h) with 100 μM MG-132. After the washing step, spermatozoa were equilibrated in CM for 15 min. The motility assessment was performed as described previously.

### 2.3. Western blotting and immunodetection

The CM was removed by centrifugation at 300 × g and spermatozoa were washed twice with phosphate-buffered saline (PBS). The supernatant was removed and 100 μl of 2 × concentrated Laemmli sample buffer [20% (v/v) glycerol, 4% (w/v) Sodium Dodecyl Sulfate (SDS), 0.125 M Tris-HCl, pH 6.8, 0.005% (w/v) bromophenol blue] was added to 50 μl sperm pellets (5 × 10^7^ cells). Spermatozoa were lysed on ice and stirred for 5 min over the course of 1 h. Then the samples were boiled for 5 min and centrifuged at 10,000 × g for 5 min.

The Mini-PROTEAN Tetra system (Bio-Rad, CA, USA) was used. Proteins were separated in a 12% polyacrylamide separating gel (12% (w/v) Acrylamide / Bis-acrylamide solution (Bio-Rad); 1.5 M Tris-HCl (Bio-Rad), pH 8.8; 0.1% (w/v) SDS; TEMED; 0.1% (w/v) ammonium persulfate) and 4% stacking gel (4% Acrylamide/Bis-Acrylamide solution; 0.5 M Tris-HCl pH 6.8 (Bio-Rad); 0.1% SDS; TEMED; 0.1% ammonium persulfate). Precision Plus Protein Dual Color Standards (Bio-Rad) were used to estimate the molecular weights of resolved sperm proteins. The separated proteins were transferred to a nitrocellulose membrane (GE Healthcare Life Science, Sweden) for 1.5 h at 0.5 A. The transferred proteins were visualized with Ponceau S. The membranes were blocked with 5% Blotto non-fat dry milk (Chem Cruz, Santa Cruz Biotechnology, Inc., TX, USA) dissolved in PBS for 1 h at room temperature. The membranes were afterward incubated with mouse anti-phosphotyrosine antibody (clone 4G10; Millipore, MA, USA) diluted 1:1,000 in PBS or mouse anti-ubiquitin antibody (FK2, 1:250 dilution, monoclonal mouse antibody recognizing mono- and polyubiquitinated conjugates; ENZO Life Sciences, NY, USA) at 4°C overnight. After incubation, the membranes were washed 3 × in PBS with 0.1% (v/v) Tween-20 (PBS-T) for 10 min. Next, the membranes were incubated with an appropriate species-specific secondary antibody (goat anti-mouse IgG, Bio-Rad, 1:3,000 in PBS) for 1 h at room temperature on a rolling platform. The membranes were washed 5 × with PBS-T for 5 min and then in PBS for 5 min. Subsequently, membranes were reacted with a chemiluminescent substrate (ThermoFisher Scientific, MA, USA), and specific protein bands recognized by the antibodies used were imaged with an AZURE Biosystems C300 instrument (Azure Biosystems, CA, USA). The densitometric analysis was performed in IMAGE Studio Digits Ver 3.1 (LI-COR Biotechnology, NE, USA).

### 2.4. Image-based flow cytometry

During sperm IVC (see sperm *in vitro* capacitation), 1 mL was taken from each sample at time zero and hourly intervals. Spermatozoa were pelleted by centrifugation at 300 × g for 10 min, washed twice with PBS, and fixed with 100 μL of 2% (w/v) formaldehyde in dH_2_O for 20 min for ubiquitinated proteins detection. Following the fixation step, spermatozoa were washed with PBS, and centrifuged at 300 × g, 4°C, for 5 min; and 100 μL of Superblock in PBS (ThermoFisher Scientific) was added and incubated for 15 min. Subsequently, the samples were washed twice with PBS and by centrifugation at 300 × g for 5 min. Spermatozoa were incubated with a mouse anti-ubiquitin antibody (1:10 in PBS, Ubi-1, Novus Biologicals, Englewood, CO, USA) for 2 h at room temperature. After the incubation, 100 μL of PBS was added and spermatozoa were washed twice with PBS by centrifugation at 300 × g for 5 min. 50 μL of a secondary antibody [1:300 in PBS, Goat anti-Mouse IgG (H + L) Highly Cross-Adsorbed conjugated to Alexa Fluor^®^ Plus 488; Invitrogen, CA, USA] was added and the samples were incubated for 1 h at room temperature. After the incubation, 100 μL of PBS was added, and the samples were centrifuged at 300 × g for 5 min. Subsequently, 50 μL of PNA lectin conjugated with rhodamine (1:500 in PBS, Vector Laboratories, Newark, CA, USA) was added to the pellet for the acrosome staining and the samples were incubated for 15 min at room temperature. After incubation, 100 μL of PBS was added, and the samples were centrifuged at 300 × g for 5 min. 50 μL of DAPI aqueous solution (1:1,000, Invitrogen) was added to each sample.

Image-based flow cytometry (IBFC) was then performed using Amnis^®^ Flow ImageStream^®^XMark II (AMNIS Luminex Corporation, TX, USA). The following instrument settings were used: objective 40 ×, first and second camera gain was 1, flow core diameter and speed were 10 μm and 66 mm.s^−1^, 488 nm laser (intensity 70 mW), 405 nm laser (intensity 50 mW) bright field with two LEDs (40 and 64 mW). Signals were observed in four channels: Ch02 (488 nm) for AlexaFluor 488, Ch03 (561 nm) for rhodamine, Ch07 (405 nm) for DAPI, and Ch09 for bright field. At least 5,000 events were collected per sample. The raw data collection was done using INSPIRE^®^ software (AMNIS Luminex Corporation, Austin, TX, USA). Subsequent analyses were done in IDEAS software Version 6.0 software (AMNIS Luminex Corporation). The gating strategy for single-cell events is included in the Supplementary material.

### 2.5. Determination of sperm viability by conventional flow cytometry

50 μL (2.5 × 10^6^ sperm) of each sperm treatment group was collected at times 0, 1, 2, and 3 h. Samples were washed three times at 300 × g for 5 min with PBS. Subsequently, Zombie UV^TM^ fixable viability kit (#423107, Biolegend, CA, USA) was used according to the manufacturer's protocol. The samples were fixed for 20 min with 50 μL of 4% (v/v) formaldehyde with 2% BSA. Spermatozoa were centrifuged at 300 × g for 5 min and resuspended in the original volume of PBS. Sperm samples were analyzed using BD LSRFortessa TM SORP instrument (Becton Dickinson, CA, USA) with a 405 nm laser line excitation (50 mW) and a 450/50 nm emission filter. Zombie UV^TM^ negative spermatozoa were considered viable.

### 2.6. Data and statistical analysis

Unless otherwise stated, all statistical analyses were performed in GraphPad Prism 9.0 (GraphPad Sofware, CA, USA).

Data of (i) percentages of totally and progressively motile spermatozoa; (ii) individual kinematic parameters; (iii) sperm viability; (iv) IBFC and finally densitometry was analyzed by Kruskal–Wallis non-parametric ANOVA. The measured values of the experimental and control groups were compared with each other at the same time points. Statistical significance were determined by Dunn's *post-hoc* test. Western blot immunodetection experiments were performed in five independent replicates. Densitometric analysis was accomplished in Image Studio Digits software (LI-COR Biosciences, NE, USA).

To identify sperm sub-populations individual sperm kinematic parameters from CASA were processed by k-mean cluster analysis in STATISTICA 12 (StatSoft, Czech Republic). Euclidean distances algorithm processed variables LIN, STR, ALH, VCL, VSL, and VAP with 5 iterations were used to define three clusters (sub-populations). According to computed means of selected variables, individual spermatozoon was afterward assigned to one of three specific sperm subpopulations: fast, medium fast, and slow locally motile (Table 1). To precisely extract the hyperactivated sperm population *k*-mean cluster analysis coupled with Principal Component Analysis (PCA) was used. PCA was used for the reduction of the number of variables (ALH, VAP, and STR) which will enter for subsequent cluster analysis. The selection of variables was based on the matrix of components. Thereby, the aforementioned variables have been selected based on the highest values in the matrix of components. The further step was the standardization of selected values (mean = 0 and SD = 1). This processing was done according to the study (34). Statistical differences were analyzed between groups of capacitated, vehicle control, and experimental groups.

**Table 1:** Characterization of different sperm subpopulations determined by cluster analysis of kinematic parameters of motile spermatozoa.

**Cluster**	**LIN (%)**	**STR (%)**	**ALH (μm)**	**VAP (μm.s^−1^)**	**VSL (μm.s^−1^)**	**VCL (μm.s^−1^)**
Fast progressive motile sperm	56.39 ± 30.20	89.01 ± 15.1	5.59 ± 2.3	103.29 ± 22.17	92.83 ± 28.25	178.07 ± 37.14
Medium motile sperm	44.51 ± 21.42	80.18 ± 211	4.6 ± 1.9	62.89 ± 14.06	50.66 ± 18.36	121.41 ± 25.05
Slow locally motile sperm	34.60 ± 17.68	75.27 ± 23.3	2.9 ± 1.63	30.69 ± 11.15	23.22 ± 10.78	70.37 ± 19.97

ALH, amplitude of lateral head displacement; LIN, linearity; STR, straightness; VAP, average path velocity; VCL, curvilinear line velocity; VSL, straight line velocity.

## 3. Results

### 3.1. Sperm motility parameters change during *in vitro* capacitation in proteasomally-inhibited spermatozoa

**Figure 1:**
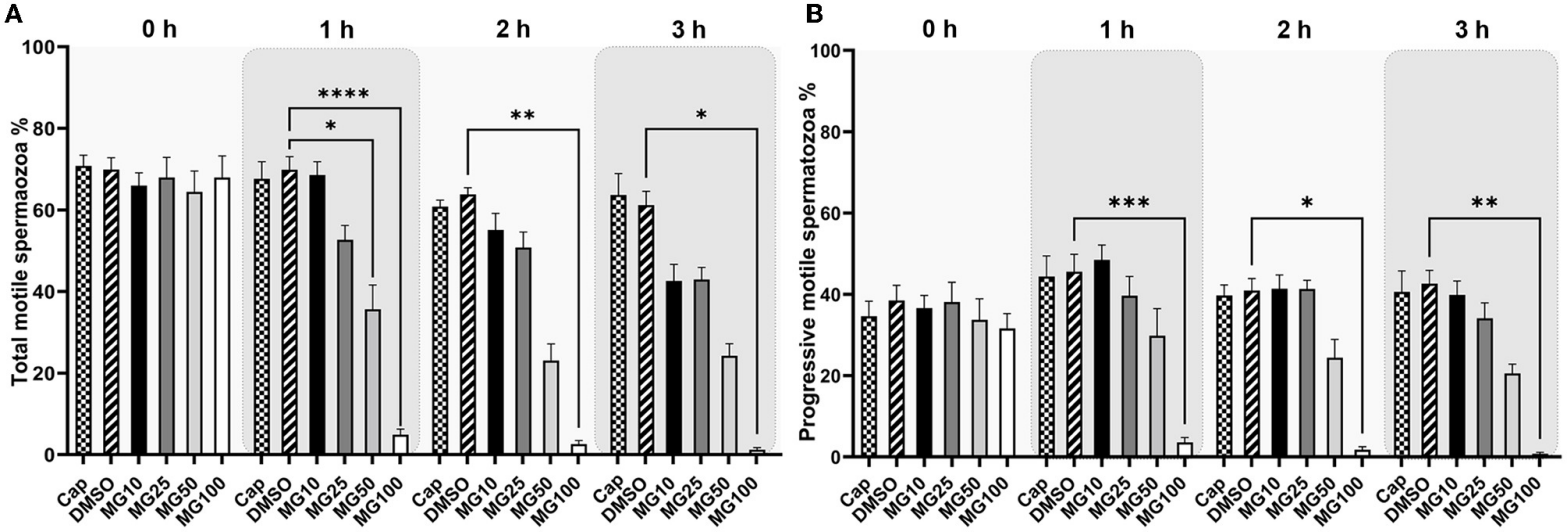
Total **(A)** and progressive **(B)** sperm motility evaluation during sperm *in vitro* capacitation (IVC) under proteasomal inhibition by ascending concentrations of MG-132 (10, 25, 50, and 100 μM). Control treatment groups comprised IVC spermatozoa incubated (i) without both the inhibitor and vehicle (Cap) and (ii) without the inhibitor and with the vehicle (DMSO). Data are presented as mean ± SEM. Significance levels *p ≤ 0.05, **p ≤ 0.01, ***p ≤ 0.001, ****p ≤ 0.0001; *n* = 20.

Total and progressive sperm motility as well as individual kinematic parameters during IVC with various proteasomal inhibiting conditions were measured by the CASA system. The effect of proteasomal inhibition on sperm motility during IVC is presented in Figure 1. A significant decrease in total and progressive motility was observed after 1-, 2-, and 3-h IVC in the treatment group with the highest MG-132 concentration (MG100) vs. IVC in the absence of inhibitor (vehicle control DMSO) (*p* ≤ 0.0001 after the 1-h incubation; *p* ≤ 0.01, *p* ≤ 0.05 after 2 and 3 h respectively). Furthermore, a significant decrease (*p* ≤ 0.05) in total sperm motility was observed after **1**-h IVC in the presence of 50 μM MG-132. Reversibility of the MG-132 treatment is documented by the significantly restored sperm motility (*p* ≤ 0.05) (see Supplementary Videos S1, S2, Supplementary Figure S1); after a 2-h incubation of spermatozoa with the inhibitor the highest concentration of 100 μM, and subsequent washing, the recovery of sperm motility was observed.

The effect of proteasomal inhibition on individual kinematic parameters of sperm motility during IVC is shown in Table 2 and Supplementary Figure S2. Our results showed similar values of linearity (LIN) and straightness (STR) confirming the relation between these two parameters. All three parameters (LIN, ALH, STR) increased during the course of sperm IVC in all treatment groups. At time 0, LIN significantly decreased (*p* ≤ 0.05) in the MG10 and MG25 groups in comparison to vehicle control (DMSO). After 1-h sperm IVC, the LIN trend remained consistent for MG10 and MG25, but an opposite pattern was observed for MG50. After **2** and **3** h of sperm IVC, all MG-132 concentrations a significant increase (*p* ≤ 0.05).

**Table 2:** Kinematic parameters evaluation during sperm *in vitro* capacitation (IVC) under proteasomal inhibition by MG-132 at various concentrations (10, 25, 50, and 100 μM) including and vehicle control (DMSO) control.

**Kinematic parameter**	**Experimental group**	**Time of capacitation**			
		**0 h**	**1 h**	**2 h**	**3 h**
LIN (%)	Cap	31.61 ± 0.18	48.10 ± 0.21	45.61 ± 0.32	47.36 ± 0.35
	DMSO	33.55 ± 0.14	43.33 ± 0.19	42.30 ± 0.24	45.41 ± 0.29
	MG10	**31.39** ±0.17^*^	**44.99** ±0.22^*^	**48.91** ±0.31^*^	**50.58** ±0.39^*^
	MG25	34.70 ± 0.21	**44.28** ±0.31^*^	**49.61** ±0.35^*^	**53.30** ±0.33^*^
	MG50	33.77 ± 0.23	**49.08** ±0.42^*^	**53.18** ±0.37^*^	**53.75** ±0.47^*^
	MG100	**32.12** ±0.22^*^	**45.50** ±0.87^*^	**47.02** ±1.49^*^	**49.14** ±1.24^*^
STR (%)	Cap	71.89 ± 0.30	82.86 ± 0.19	79.75 ± 0.32	81.25 ± 0.33
	DMSO	76.52 ± 0.24	82.52 ± 0.18	80.18 ± 0.25	82.36 ± 0.28
	MG10	**73.02** ±0.30^*^	81.93 ± 0.22	**83.73** ±0.28^*^	**86.89** ±0.32^*^
	MG25	75.73 ± 0.28	82.74 ± 0.30	**84.92** ±0.29^*^	**87.64** ±0.28^*^
	MG50	75.34 ± 0.35	**85.68** ±0.37^*^	**87.20** ±0.33^*^	**86.68** ±0.41^*^
	MG100	**73.01** ±0.32^*^	**81.29** ±0.97^*^	81.47 ± 1.68	84.91 ± 1.27
ALH (μm)	Cap	4.64 ± 0.03	3.72 ± 0.02	3.76 ± 0.03	4.11 ± 0.03
	DMSO	4.02 ± 0.02	3.38 ± 0.02	3.76 ± 0.02	4.01 ± 0.03
	MG10	4.42 ± 0.03	**4.12** ±0.03^*^	**4.25** ±0.03^*^	3.97 ± 0.03
	MG25	**4.12** ±0.03^*^	**3.79** ±0.03^*^	**3.90** ±0.03^*^	**4.22** ±0.03^*^
	MG50	**3.90** ±0.03^*^	**3.70** ±0.04^*^	**4.31** ±0.04^*^	4.06 ± 0.04
	MG100	**3.84** ±0.03^*^	3.35 ± 0.07	3.51 ± 0.12	**3.43** ±0.12^*^
VSL (μm/s)	Cap	37.85 ± 0.30	58.77 ± 0.37	49.54 ± 0.48	52.17 ± 0.53
	DMSO	35.84 ± 0.24	44.98 ± 0.29	42.99 ± 0.35	49.06 ± 0.45
	MG10	34.93 ± 0.28	**52.10** ±0.38^*^	**59.27** ±0.52^*^	**54.97** ±0.62^*^
	MG25	**39.12** ±0.35^*^	46.61 ± 0.51	**54.37** ±0.53^*^	**55.70** ±0.53^*^
	MG50	36.06 ± 0.39	**47.00** ±0.63^*^	**51.78** ±0.56^*^	**50.02** ±0.65^*^
	MG100	**32.46** ±0.32^*^	**35.69** ±1.02^*^	**31.50** ±1.66^*^	**32.25** ±1.39^*^
VCL (μm/s)	Cap	113.75 ± 0.70	119.36 ± 0.50	106.26 ± 0.64	109.01 ± 0.68
	DMSO	106.54 ± 0.50	102.10 ± 0.42	99.82 ± 0.52	105.76 ± 0.60
	MG10	**111.63** ±0.63^*^	**112.64** ±0.55^*^	**117.89** ±0.68^*^	106.46 ± 0.85
	MG25	**112.85** ±0.64^*^	101.77 ± 0.74	108.88 ± 0.75	105.46 ± 0.80
	MG50	105.89 ± 0.72	**92.48** ±0.87^*^	96.48 ± 0.80	**93.99** ±0.97^*^
	MG100	**102.05** ±0.61^*^	**77.71** ±1.62^*^	**67.25** ±2.66^*^	**67.46** ±2.63^*^
VAP (μm/s)	Cap	53.62 ± 0.36	67.83 ± 0.36	58.79 ± 0.45	61.12 ± 0.49
	DMSO	46.54 ± 0.26	52.38 ± 0.28	51.22 ± 0.34	56.99 ± 0.43
	MG10	**48.15** ±0.32^*^	**61.17** ±0.37^*^	**67.36** ±0.49^*^	**60.81** ±0.60^*^
	MG25	**51.04** ±0.37^*^	53.93 ± 0.49	**61.32** ±0.51^*^	**61.68** ±0.51^*^
	MG50	47.55 ± 0.42	52.93 ± 0.62	**57.68** ±0.54^*^	56.02 ± 0.62
	MG100	**44.14** ±0.35^*^	**42.16** ±0.99^*^	**37.40** ±1.61^*^	**37.80** ±1.49^*^

Significances of vehicle control (DMSO) vs. experimental groups with MG-132 inhibitor in individual time of IVC. Data are presented as mean ± SEM. Significance level ^*^p ≤ 0.05; n = 20. MG10, 10 μM MG-132; MG25, 25 μM MG-132; MG50, 50 μM MG-132; MG100, 100 μM MG-132; LIN, linearity; STR, straightness; ALH, amplitude of lateral head displacement; VSL, straight line velocity; VAP, average path velocity; VCL, curvilinear line velocity. Bold value indicates as they are significantly different.

At time 0, a significant decrease (*p* ≤ 0.05) of sperm lateral head displacement (ALH) was observed in groups MG25, MG50, and MG100 *vs*. vehicle control (DMSO). After 1 and 2 h of sperm IVC, ALH was significantly higher (*p* ≤ 0.05) in MG10, MG25, and MG50 treatment groups when compared vehicle control (DMSO). After **3**-h of IVC, significant differences were observed between MG25, and MG100 *vs*. DMSO control. At the onset of IVC, straightness (STR) was significantly affected for inhibitor concentrations 10 and 100 vehicle control group (*p* ≤ 0.05). After the second and third hour of capacitation, there was a significant increase in experimental groups MG10, MG25 and MG50 compared to vehicle control (DMSO).

Straight-line velocity (VSL) was significantly decreased (*p* ≤ 0.05) in MG25 and MG100 groups at the beginning of incubation. This pattern remained partially consistent after 1-h sperm IVC with the most substantial decrease in the MG100 group. Additional 1 and 2 h of incubation resulted in a significant increase in VSL in all experimental groups (*p* ≤ 0.05) compared to vehicle control (DMSO).

For curvilinear velocity (VCL), results indicate a significant increase for all inhibitor-treated groups (*p* ≤ 0.05) at time 0 except MG100 when compared to the vehicle control (DMSO). However, this was changed during the course of sperm IVC, when a significant decrease in VCL (*p* ≤ 0.05) in all inhibitor-treated groups except MG10 was observed. After 1, 2 h of sperm IVC, a significant increase was observed in the MG10 group (*p* ≤ 0.05), while a significant decrease was observed in MG50 and MG100 (*p* ≤ 0.05) in relation to vehicle control (DMSO).

The addition of MG-132 in the highest concentration (MG100), resulted in a significant decrease (*p* ≤ 0.05) in the average velocity path (VAP) for all incubation times. Interestingly the group MG10 showed a significant (*p* ≤ 0.05) increment in the values of this sperm kinematic parameter throughout the whole incubation period. After 2 h of sperm IVC, a significant VAP increase occurred in the MG10, MG25 and MG50 (*p* ≤ 0.05) groups when compared DMSO control.

**Figure 2:**
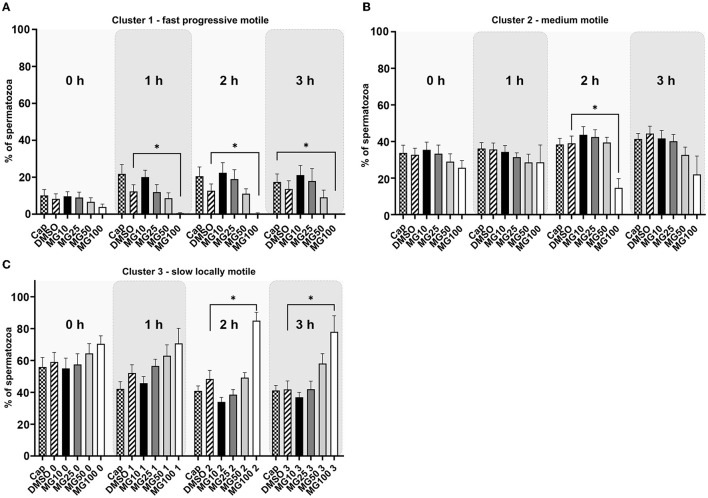
K-means cluster analysis evaluation of sperm motility based on the cluster distribution throughout all kinematic parameters during sperm *in vitro* capacitation with MG-132 proteasomal inhibition (10, 25, 50, and 100 μM) including capacitating (Cap) and vehicle (DMSO) controls. Data are presented as mean ± SEM. Significance levels *p ≤ 0.05. **(A)** Cluster 1 with rapid progressively motile spermatozoa, **(B)** Cluster 2 with medium level progressive motile spermatozoa, **(C)** Cluster 3 with low motile, static spermatozoa.

The *k*-means cluster analysis was performed to partition spermatozoa into three subpopulations (clusters) in regard to their combined kinematic parameters (Figure 2). In the progressively motile sperm cluster (cluster 1; Figure 2A), an expected change in sperm motility redistribution toward fast progressive motility was observed during the course of sperm IVC. The redistribution toward the fast progressive motility was significantly reduced when spermatozoa were capacitated with 100 μM MG-132 after 1, 2, and 3 h of incubation (*p* ≤ 0.05) when compared to vehicle control (DMSO). This pattern was reflected in the distribution of sperm motility in cluster 3 (Figure 2C), wherein a significant increase of slow, locally motile spermatozoa was observed after 2 and 3 h of IVC in the MG100 experimental group (*p* ≤ 0.05) compared to vehicle control (DMSO). In the medium-level motile sperm cluster (cluster 2; Figure 2B), significance in the motility redistribution was observed among vehicle control (DMSO) and the experimental group with the highest concentration of inhibitor MG-132 after 2 h of incubation.

**Figure 3:**
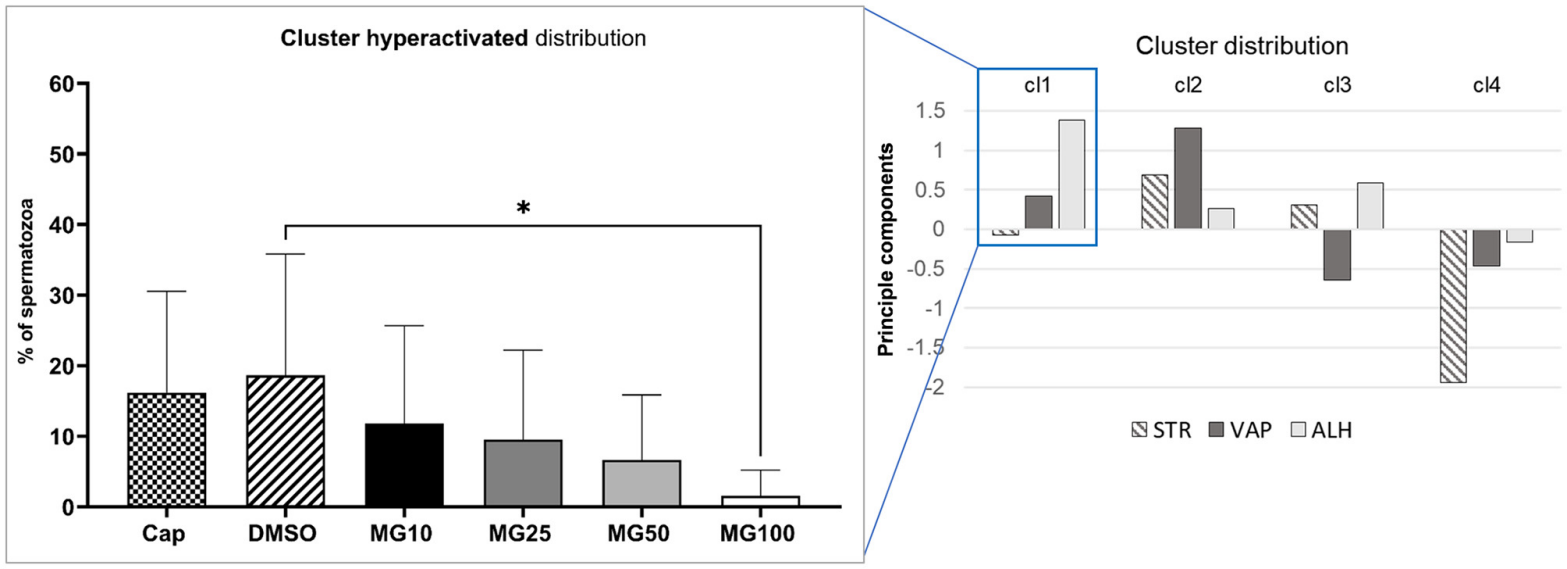
Evaluation of hyperactivated sperm motility based on the cluster distribution of three kinematic parameters (STR, VAP, and ALH). Sperm kinematic parameters were measured during IVC under proteasomal inhibition by MG-132 at various concentrations (10, 25, 50, and 100 μM), including capacitating (Cap) and vehicle (DMSO) controls. The right panel represents cluster identification based on cluster analysis coupled with PCA, followed by further data standardization. The left panel represents the effect of the MG-132 inhibitor on the hyperactivated cluster. Data are presented as mean ± SEM. Significance level *p ≤ 0.05.

Principal component analysis coupled with k-mean clustering analysis was performed in order to reveal potential subtle changes in the hyperactivated sperm subpopulations caused by proteasomal inhibition. Results presented in Figure 3 clearly show a significant decrease in sperm distribution (*p* ≤ 0.05) under the strongest (MG100) inhibiting conditions.

**Figure 4:**
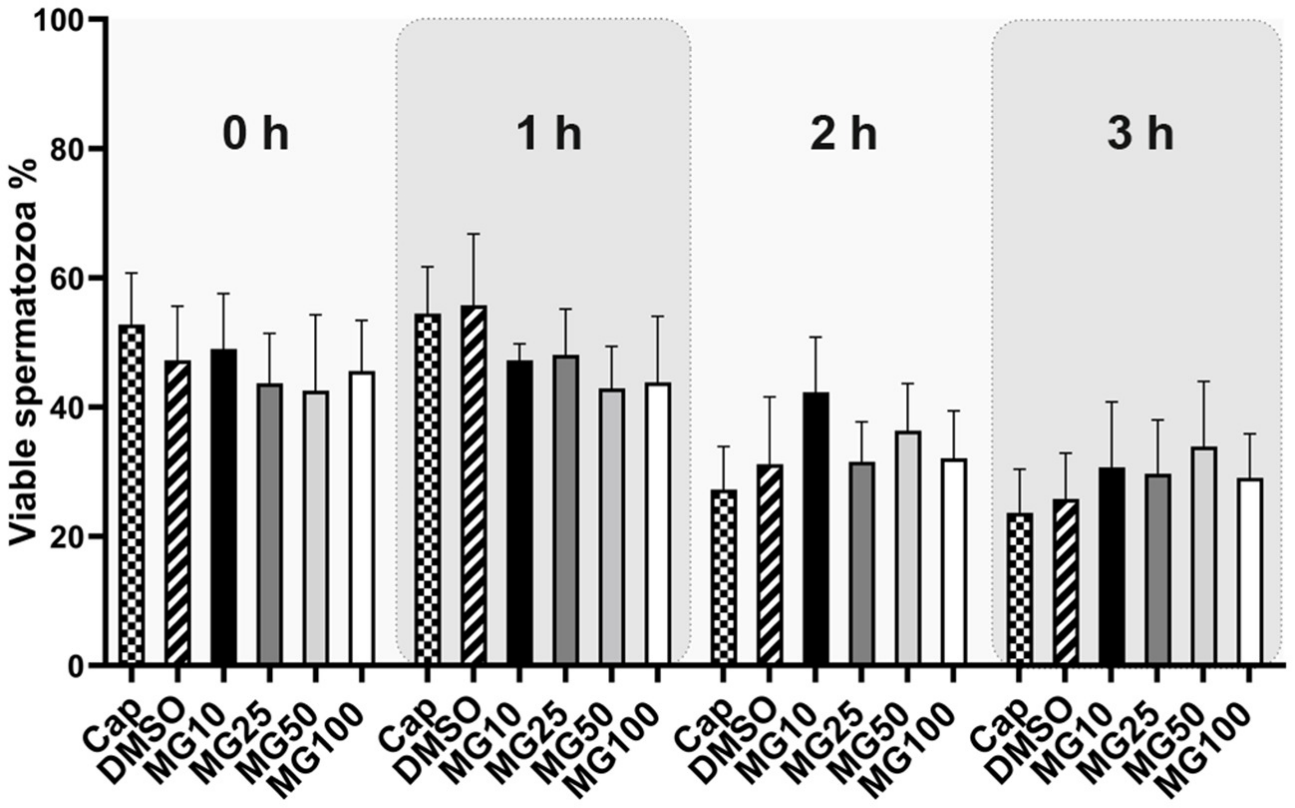
Sperm viability assessment during 3 h of *in vitro* capacitation (IVC) under proteasomal inhibition by MG-132 of various concentrations (10, 25, 50, and 100 μM). Control treatment groups comprised IVC spermatozoa incubated (i) without both the inhibitor and vehicle (Cap) and (ii) without the inhibitor and with the vehicle (DMSO). Data are presented as mean ± SEM. No significant differences were observed; *n* = 20.

Sperm viability was assessed by conventional flow cytometry using Zombie UV^TM^ fixable viability kit and is expressed as a percentage of viable spermatozoa (Zombie UV^TM^ negative). The sperm viability status, during sperm IVC in all treatment groups, is presented in Figure 4 together with the time lapse assessment of the inhibitor effect during IVC (Supplementary Figure S3). The percentage of viable spermatozoa was not affected by MG-132 (*p* > 0.05) regardless of MG-132 concentration, when compared to control groups (Cap, DMSO).

### 3.2. Protein tyrosine phosphorylation oscillations in spermatozoa during *in vitro* capacitation

**Figure 5:**
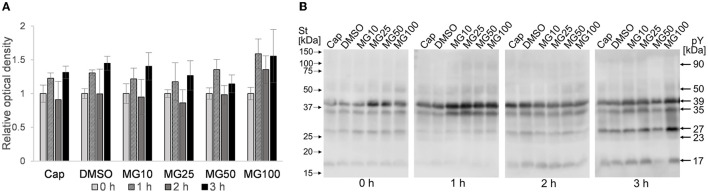
Detection of total phosphotyrosine levels in sperm protein extracts during sperm *in vitro* capacitation under proteasomal inhibiting conditions at various concentrations of MG-132 (10, 25, 50, and 100 μM) including capacitating (Cap) and vehicle (DMSO) controls. **(A)** Representative Western blot shows the molecular masses of pY. **(B)** The graph represents the total pY determined by densitometric analysis. Data are presented as mean ± SEM. No significant differences were observed; *n* = 5.

A level of sperm protein pY was monitored during the course of sperm IVC under various proteasome-inhibiting conditions, including capacitating and vehicle controls (Figure 5). Total pY was assessed with WB detection of phosphorylated proteins in sperm lysates of all experimental groups, with predominantly detected proteins of 17, 23, 27, 35, 39, 50, and 90 kDa (Figure 5B). Densitometric analysis showed that the abundance of pY increased after the first hour of sperm IVC, followed by a decrease after **2** h, and ending in a repeated increase of pY after **3** h of sperm IVC for all treatment groups. No significant trend (*p* > 0.05) in the level of pY was observed during the course of sperm IVC as well as in the treatment groups, contributed by high sample-to-sample variability (Figure 5A).

### 3.3. The sperm ubiquitination level does not change with proteasome inhibition during sperm *in vitro* capacitation

**Figure 6:**
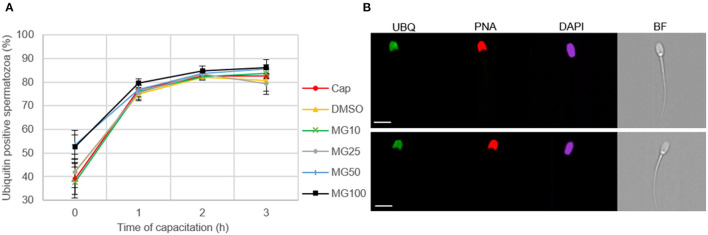
Assessment of sperm ubiquitination during sperm *in vitro* capacitation (IVC) under proteasome inhibiting conditions at various concentrations of MG-132 (10, 25, 50, and 100 μM) including capacitating (Cap) and vehicle (DMSO) controls. **(A)** Quantification of the ubiquitin-positive spermatozoa in individual treatment groups during sperm IVC. **(B)** Representative IBFC images of ubiquitin immunolabeling (UBQ) in the sperm head with antibody, acrosome status with PNA lectin and DAPI staining of the sperm nuclei; scale bar 10 μm. The images are representative of all groups. Data are presented as mean ± SEM. No significant differences were observed; *n* = 3.

Image-based flow cytometry was used to reveal changes in the total sperm ubiquitination determined by immunofluorescence staining of spermatozoa (Figure 6). The general trend, observed in all experimental groups, was that the level of sperm ubiquitination was distinctly increased after 1 h of IVC. In the MG50 and MG100 treatment groups, the mean values of total ubiquitination were higher when compared to non-inhibited control at the beginning of sperm IVC (0 h), but this is not significant. After **3**-h sperm IVC, the trend showing the highest ubiquitination level in the MG50 and MG100 groups was not significant. No significant differences were observed in the sperm total ubiquitination levels during the course of sperm IVC as well as between the treatment groups (Figure 6A). Immunofluorescence staining with an anti-ubiquitin antibody in boar spermatozoa in Figure 6B shows acrosomal labeling in all experimental groups. Spermatozoa without acrosome (PNA negative) representing only a low percentage in all groups were also negative for the anti-ubiquitin antibody.

**Figure 7:**
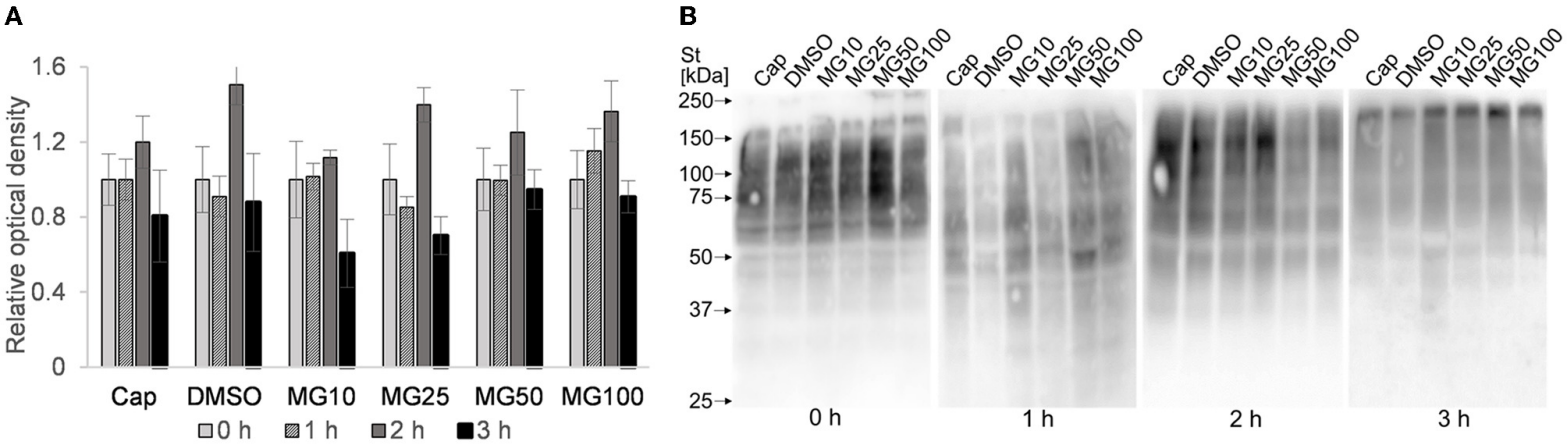
Assessment of total ubiquitination in sperm protein extracts during sperm *in vitro* capacitation (IVC) under proteasomal inhibition at various concentrations of MG-132 (10, 25, 50, and 100 μM) including capacitating (Cap) and vehicle (DMSO) controls. **(A)** The graph represents total ubiquitinated proteins determined by densitometric analysis. Data are presented as mean ± SEM. No significant differences were observed. **(B)** Representative Western blot detection of ubiquitinated proteins; *n* = 5.

In addition to IBFC, the total amount of ubiquitinated proteins was monitored in sperm lysates at hourly intervals during sperm IVC. No effect was observed between MG-132-inhibited groups and vehicle control (DMSO) during the sperm IVC. The results suggest the highest relative abundance of total sperm ubiquitination was reached after **2** h of sperm IVC regardless of the treatment (Figure 7A). The highest abundance of ubiquitinated proteins was detected in the molecular weight range from 55 to 150 kDa (Figure 7B).

## 4. Discussion

Although recent studies have shown that UPS is involved in many aspects of sperm capacitation, reviewed in Kerns et al. (35), a substantial piece of knowledge on the proteasomal regulation of sperm motility hyperactivation remains undisclosed. Our study is the first one to shed some light on the physiological role of the proteasome in boar sperm hyperactivated motility during sperm capacitation. For our experiments, we used fresh ejaculates as opposed to extended ones due to the presence of calcium and bicarbonate ions in commercial extenders that were shown to initiate capacitation-related events (36). We demonstrated that the addition of the proteasomal inhibitor MG-132 to the capacitation medium significantly suppressed total and progressive motility in a dose-dependent manner during sperm IVC. In addition to the effect of proteasomal inhibition during sperm IVC on total and progressive motility, we are also documenting the effect of proteasome inhibition on the hyperactivated sperm population redistribution in the MG-132 treated groups. As the subjective assessment of hyperactivated sperm motility is very difficult, computer-assisted sperm analysis (CASA) represents a useful tool for the objective assessment of sperm motility (27). Furthermore, the exact movement pattern of hyperactivated spermatozoa is difficult to define as it varies among species, due to species differences in the thickness and length of a flagellum, and the physical environment in which rheotaxis occurs (24). As trajectories of motile spermatozoa are complex, there is no single kinematic parameter that would reflect hyperactivated motility reliably (27). To mitigate these challenges, we used a cluster analysis based on a multiparametric evaluation. We confirmed that the distribution of spermatozoa between individual sperm motility clusters changed during the course of sperm IVC, and we also demonstrated a dependency of sperm redistribution on the proteasomal inhibitor concentration, in a dose-dependent manner. The abundance of spermatozoa decreased proportionally with an increasing inhibitor concentration and capacitation time in spermatozoa with rapid progressive motility and medium-level progressive motility cluster. The said decrease was reflected in a proportional abundance increase of spermatozoa in the low-motile sperm cluster. Moreover, for distribution analysis of the hyperactivated sperm cluster principal component analysis was utilized prior to k-means clustering analysis based on the study by Ibanescu et al. (34). With this narrowing of kinematic parameter spectrum (STR, ALH, VAP) entering into the cluster analysis, a subpopulation of hyperactivated spermatozoa was successfully extracted. In this design, lateral head displacement (ALH) was included as the main distinguishing parameter for the increment of flagellar curve amplitude which is related to sperm hyperactivation (37). Even in this case, there was a clear effect of dose-dependent proteasomal inhibition on the hyperactivated sperm subpopulation abundance. We observed a significant decrease in the hyperactivated sperm subpopulation abundance in the experimental group with the highest MG-132 concentration (100 μM) when compared to the non-inhibited control group.

The trend (not significant) in a viability decrease during the first 3 h of incubation is clearly visible. Interestingly for viability, there is no significant decline in the population of spermatozoa showing non-viable status in experimental groups with the highest concentration of inhibitor, as confirmed by analysis of total and progressive motility. The reversible nature of motility inhibition (see Supplementary Video S1) and no significant difference in sperm viability between the treatment groups exclude the possibility that motility inhibition might have been caused by a disruption of sperm plasma membrane integrity.

Sperm motility is strictly regulated in a response to the environment by subsequent changes in energy requirements during their passage through the female reproductive tract. The direct mechanistic link between the energy demand and energy supply is represented by the AMP-activated protein kinase (AMPK) (38–40). The AMPK is present in the acrosome and the midpiece of boar spermatozoa (41, 42). Dysregulation of AMPK activity had an adverse effect on sperm kinematic parameters such as VAP, VSL, and VCL (41, 43). Interestingly, Ronnebaum et al. (44) reported that UPS can also regulate AMPK activity. Their study demonstrated that AMPK subunits α2, β1, and β2 are polyubiquitinated and turned-over by UPS. We hypothesize that energetic metabolism modulation through the AMPK pathway might be a possible mechanism for how UPS may regulate sperm motility during capacitation.

Another possible mechanism of sperm motility regulation by the proteasome might be through the family of AKAP scaffold proteins located in the fibrous sheet (45, 46), a structure covering the entire principal piece of the flagellum (47). Members of the AKAP family are involved in signaling pathways regulating various cellular functions. The AKAP-mediated sequestration of signal-transduction enzymes in a specific subcellular compartment ensures the proximity of the enzyme to its substrates but also segregates its activity to prevent random substrate phosphorylation. An important role of AKAPs is also their ability to form multiprotein complexes that integrate cAMP signaling with other pathways and their respective signaling events (48, 49). Several AKAPs have been reported in the sperm flagellum including AKAP1 (50), AKAP3 (51), AKAP4 (52), and AKAP11 (53). It was shown that AKAP3 abundance is reduced during the incubation of spermatozoa under capacitation-inducing conditions. Interestingly, the study by Hillman et al. (49) reported that a decrease in AKAP3 abundance after 4 h of IVC was mitigated when incubated under proteasomal inhibition, demonstrating that AKAP3 degradation is regulated by UPS. In addition, AKAP3 degradation seems to be specific and has a physiological relevance in capacitation, since another AKAP family member—AKAP8 is not degraded during IVC (49). There remains a question of how to explain the decline in total and progressive motility, and the subpopulation of hyperactivated spermatozoa when capacitated under proteasomally-inhibiting conditions.

Inhibiting the proteasomal degradation of AKAP3, as an important and specific mediator of the correct course of the sperm capacitation process (28, 49, 54, 55), can lead to incorrect targeting of key regulatory molecules to specific subcellular compartments. Furthermore, these scenarios suggest a disruption in the activation of downstream signaling molecules and incorrect signaling cues. Proteasomal inhibition can also disrupt the degradation of regulatory proteins of which the removal is essential for the activation of signal transduction enzymes.

The AKAP3 proteins anchor PRKA which is involved in sperm motility modulation in hamsters, monkeys, and humans (56–58). Furthermore, in human spermatozoa, Zapata-Carmona et al. (55) showed that UPS degrades PRKA regulatory subunit 1 (PRKAR1) localized in the sperm head and tail. This represents one of the candidate regulatory mechanisms for maintaining PRKA activity. Proteasomal inhibition during IVC can result in low PRKA activity, and insufficient protein tyrosine phosphorylation may delay the onset of sperm hyperactivation. This claim is supported by their previous study (59), where the proteasomal inhibitor epoxomicin significantly inhibited the phosphorylation of PRKA. In our study, we were monitoring the total protein pY during IVC. We observed minor oscillations in pY levels during sperm IVC as we saw an increase in pY abundance after 1 h of IVC, a decline after 2 h, and regeneration of pY after 3 h of IVC. To our best knowledge, this is the first instance of such a pY pattern reported during IVC. Proteasomal inhibition did not have a significant effect on the abundance of pY status in our experimental design. Similarly, Kong et al. (18) did not find evidence that the sperm proteasome is involved in the regulation of tyrosine phosphorylation. However, a more recent study by Qu et al. (28) showed that the level of pY significantly decreased in MG-132-inhibited boar spermatozoa after 3 h of IVC. However, in their study, the authors recorded the level of pY phosphorylation only at the end of a 3-h sperm capacitation period, as opposed to the pY phosphorylation level time lapse recording in the present study.

Moreover, several studies have shown that inhibition of PRKA by H89 during sperm IVC led to a reduction in protein pY (60) and sperm motility (61), but not to inhibition of pY on AKAP3 (62). This would imply that PRKA is involved in the downregulation of sperm motility. Interestingly, the study of Vijayaraghavan et al. (63) showed that the inhibition of PRKA catalytic activity had no significant effect on basal motility. These observations suggest that the binding of the PRKA regulatory subunit to AKAP is a key regulator of sperm motility. Other studies corroborate the relationship between pY of flagellar proteins and hyperactivation. For example, treatment of non-capacitated spermatozoa with procaine or caffeine immediately resulted in an increase in intracellular calcium levels leading to sperm hyperactivation without increasing the protein tyrosine phosphorylation rate in flagellar proteins (64, 65).

As a part of our study concerned with the impact of proteasomal inhibition on sperm motility during IVC, we also focused on monitoring the total level of sperm ubiquitination. However, the time-dependent effect of different concentrations of proteasomal inhibitor MG-132 on sperm motility did not coincide with a detectable accumulation of polyubiquitinated proteins. We observed no differences based on densitometric analysis of the WB detection of total ubiquitinated sperm proteins, nor during IBFC where we monitored median fluorescent intensities of sperm total ubiquitination. No accumulation of polyubiquitinated proteins was seen even in the groups with the highest concentrations of MG-132 inhibitor compared to the control groups, Cap (without DMSO and inhibitor) and DMSO (vehicle control). Since MG132 inhibits chymotrypsin-like activity with a much lower inhibition constant when compared to caspase-like (~300 × higher) or trypsin-like (~900 × higher) activities (31), we may propose that the residual proteasomal activity attributable to trypsin-like and caspase-like core activities accounted for the observed insignificance in the protein polyubiquitination abundance between the treatment groups. Another possible explanation might lie in the presence of 20S proteasomes that do not require a polyubiquitin signal as opposed to 26S proteasomes that recognize polyubiquitinated proteins labeled for degradation. Our unpublished data also suggest that sperm 20S proteasomes are more abundant than 26S in porcine spermatozoa. No such study was done in other species, to our best knowledge. A possible strategy for a future experimental design might be the use of proteasomal inhibitor cocktails to provide a stronger inhibition of caspase-like and trypsin-like 20S activities. The experimental design of the current study prevented the application of such a strategy since the relevant 20S inhibitors, including epoxomicin or cloasto-lactacystin-β-lactone are irreversible. Rather than monitoring the accumulation of total polyubiquitinated proteins, it may be desirable to focus on individual proteasomal substrate protein level, such as the porcine DQH/BSP1, MFGE8, and ADAM5 proteins, which accumulate after proteasomal inhibition as demonstrated in our earlier studies (9, 66).

The understanding of the mechanism of UPS regulation during mammalian sperm hyperactivation warrants future studies, including the link between UPS and protein pY during sperm IVC. It is important to directly monitor the phosphorylation of specific proteins within the proteasome-mediated signaling pathway. The regulatory mechanism of the proteasome might be linked to the degradation of AKAP3, where its ubiquitination causes the uncoupling of PRKA (28). Provided such a link exists, the incomplete inhibition of motility in the experimental group with 100 μM MG132 can be reasoned that other mechanisms in the AKAP3 degradation might be involved. Calcium ions, intracellular alkalization, lack of glucose, and reduction of ATP levels could also be involved in the regulation of AKAP3 degradation (49, 54).

In conclusion, inhibition of the 20S proteasome chymotrypsin-like activity caused a reduction in the total and progressively motile sperm populations and had a negative dose-dependent impact on sperm hyperactivation. Velocity kinematic parameters during IVC were affected, as well. Importantly, proteasomal inhibition did not impair sperm plasma membrane integrity, ruling out that non-specific cytotoxicity caused the observed effects. Furthermore, proteasomal inhibition did not interfere with protein pY, a hallmark of IVC. Similarly, the accumulation of ubiquitinated proteins was not observed. A measurable effect of proteasome inhibition on sperm hyperactivation but not at the protein tyrosine phosphorylation level implies that the changes in the protein tyrosine phosphorylation profile may not be the only prerequisite for boar spermatozoa to achieve the capacitated state.

## Data availability statement

The original contributions presented in the study are included in the article/Supplementary material, further inquiries can be directed to the corresponding authors.

## Ethics statement

Ethical review and approval was not required for the animal study because this study did not involve animal intervention. Handling or keeping of animals was according to Council Directive 98/58/EC, Act No. 154/2000 Coll. and Act of the Czech National Council No. 246/1992 Coll.

## Author contributions

LH, PP, and OS designed the experiments. LH, NZ, KH, RK, and OS performed the experiments. LH, BK, MZ, PS, KK, PP, and OS analyzed the data and wrote the paper. MS, PS, and KK critically revised the manuscript. All authors have read and approved the final version of the manuscript.

## References

[1] Young WC. A study of the function of the epididymis. II. The importance of the aging process in sperm for the length of the period during which fertilizing capacity is retained by sperm isolated in the epididymis of the guinea pig. J Morphol. (1929) 48:475–91. 10.1002/jmor.1050480208

[2] Cosentino MJ Cockett AT. Structure and function of the epididymis. Urol Res. (1986) 14:229–40. 10.1007/BF00256565 3026075

[3] Chakraborty S Saha S. Understanding sperm motility mechanisms and the implication of sperm surface molecules in promoting motility. Middle East Fertil Soc J. (2022) 27:1–12. 10.1186/s43043-022-00094-7

[4] Chang MC. Fertilizing capacity of spermatozoa deposited into the fallopian tubes. Nature. (1951) 168:697–8. 10.1038/168697b0 14882325

[5] Austin CR. Observations on the penetration of the sperm into the mammalian egg. Aust J Biol Sci. (1951) 4:581–96. 10.1071/BI9510581 14895481

[6] Davis BK. Timing of fertilization in mammals: sperm cholesterol/phospholipid ratio as a determinant of the capacitation interval (interspecies correlations/sperm cholesterol efflux/acrosome reaction). Proc Natl Acad Sci. (1981) 78:7560–4. 10.1073/pnas.78.12.7560 6950397 PMC349308

[7] Suarez SS. Mammalian sperm interactions with the female reproductive tract. Cell Tissue Res. (2016) 363:185–94. 10.1007/s00441-015-2244-2 26183721 PMC4703433

[8] Sutovsky P. Sperm proteasome and fertilization. Reproduction. (2011) 142:1–14. 10.1530/REP-11-0041 21606061

[9] Zigo M Manaskova-Postlerova P Jonakova V Kerns K Sutovsky P. Compartmentalization of the proteasome-interacting proteins during sperm capacitation. Sci Rep. (2019) 9:1–18. 10.1038/s41598-019-49024-0 31467409 PMC6715765

[10] Jonsson E Htet ZM Bard JAM Dong KC Martin A. Ubiquitin modulates 26S proteasome conformational dynamics and promotes substrate degradation. Sci Adv. (2022) 8:eadd9520. 10.1126/sciadv.add9520 36563145 PMC9788759

[11] Escalier D. New insights into the assembly of the periaxonemal structures in mammalian spermatozoa. Biol Reprod. (2003) 69:373–8. 10.1095/biolreprod.103.015719 12672659

[12] Mochida K Tres LL Kierszenbaum AL. Structural features of the 26S proteasome complex isolated from rat testis and sperm tail. Mol Reprod Dev. (2000) 57:176–84. 10.1002/1098-2795(200010)57:2&lt;176::AID-MRD9&gt;3.0.CO;2-O 10984418

[13] Morozov A Karpov VL. Proteasomes and several aspects of their heterogeneity relevant to cancer. Front Oncol. (2019) 9:761. 10.3389/fonc.2019.00761 31456945 PMC6700291

[14] Kisselev AF. Site-specific proteasome inhibitors. Biomolecules. (2022) 12:54. 10.3390/biom12010054 35053202 PMC8773591

[15] Leestemaker Y Ovaa H. Tools to investigate the ubiquitin proteasome system. Drug Discov Today Technol. (2017) 26:25–31. 10.1016/j.ddtec.2017.11.006 29249239

[16] Wojcik C DeMartino GN. Intracellular localization of proteasomes. Int J Biochem Cell Biol. (2003) 35:579–89. 10.1016/S1357-2725(02)00380-1 12672451

[17] Nakamura N. Ubiquitination regulates the morphogenesis and function of sperm organelles. Cells. (2013) 2:732–50. 10.3390/cells2040732 24709878 PMC3972651

[18] Kong M Diaz ES Morales P. Participation of the human sperm proteasome in the capacitation process and its regulation by protein kinase A and tyrosine kinase. Biol Reprod. (2009) 80:1026–35. 10.1095/biolreprod.108.073924 19144957

[19] Baker MA Reeves G Hetherington L Aitken RJ. Analysis of proteomic changes associated with sperm capacitation through the combined use of IPG-strip pre-fractionation followed by RP chromatography LC-MS/MS analysis. Proteomics. (2010) 10:482–95. 10.1002/pmic.200900574 19943266

[20] Choi YJ Uhm SJ Song SJ Song H Park JK Kim T. Cytochrome C upregulation during capacitation and spontaneous acrosome reaction determines the fate of pig sperm cells: linking proteome analysis. J Reprod Dev. (2008) 54:68–83. 10.1262/jrd.19116 18094529

[21] Ecroyd HW Jones RC Aitken RJ. Endogenous redox activity in mouse spermatozoa and its role in regulating the tyrosine phosphorylation events associated with sperm capacitation. Biol Reprod. (2003) 69:347–54. 10.1095/biolreprod.102.012716 12672670

[22] Bailey JL. Factors regulating sperm capacitation. Syst Biol Reprod Med. (2010) 56:334–48. 10.3109/19396368.2010.512377 20849222

[23] Leemans B Stout TAE de Schauwer C Heras S Nelis H Hoogewijs M. Update on mammalian sperm capacitation: how much does the horse differ from other species? Reproduction. (2019) 157:R181–97. 10.1530/REP-18-0541 30721132

[24] Suarez SS. Hyperactivated motility in sperm. J Androl. (1996) 17:331–5. 10.1002/j.1939-4640.1996.tb01797.x 8889694

[25] Yanagimachi R. Fertility of mammalian spermatozoa: its development and relativity. Zygote. (1994) 2:371–2. 10.1017/S0967199400002240 8665172

[26] Mortimer ST. CASA: practical aspects. J Androl. (2000) 21:515–24. 10.1002/J.1939-4640.2000.TB02116.X 10901437

[27] Mortimer ST de Jonge CJ. CASA-computer-aided sperm analysis. Encycl Reprod. (2018) 12:59–63. 10.1016/B978-0-12-801238-3.64935-8

[28] Qu X Han Y Chen X Lv Y Zhang Y Cao L. Inhibition of 26S proteasome enhances AKAP3-mediated cAMP-PKA signaling during boar sperm capacitation. Anim Reprod Sci. (2022) 247:107079. 10.1016/j.anireprosci.2022.107079 36209601

[29] Glickman MH Ciechanover A. The ubiquitin-proteasome proteolytic pathway: destruction for the sake of construction. Physiol Rev. (2002) 82:373–428. 10.1152/physrev.00027.2001 11917093

[30] Morales P Diaz E Kong M. Proteasome activity and its relationship with protein phosphorylation during capacitation and acrosome reaction in human spermatozoa. Soc Reprod Fertil Suppl. (2007) 65:269–73. 17644968

[31] Kisselev AF Goldberg AL. Proteasome inhibitors: from research tools to drug candidates. Chem Biol. (2001) 8:739–58. 10.1016/S1074-5521(01)00056-4 11514224

[32] Kisselev AF Callard A Goldberg AL. Importance of the different proteolytic sites of the proteasome and the efficacy of inhibitors varies with the protein substrate. J Biol Chem. (2006) 281:8582–90. 10.1074/jbc.M509043200 16455650

[33] Rock KL Gramm C Rothstein L Clark K Stein R Dick L. Inhibitors of the proteasome block the degradation of most cell proteins and the generation of peptides presented on MHC class I molecules. Cell. (1994) 78:761–71. 10.1016/S0092-8674(94)90462-6 8087844

[34] Ibanescu I Leiding C Bollwein H. Cluster analysis reveals seasonal variation of sperm subpopulations in extended boar semen. J Reprod Dev. (2018) 64:33–9. 10.1262/jrd.2017-083 29081440 PMC5830356

[35] Kerns K Morales P Sutovsky P. Regulation of sperm capacitation by the 26S proteasome: an emerging new paradigm in spermatology. Biol Reprod. (2016) 94:117–8. 10.1095/biolreprod.115.136622 27053366

[36] Dube C Beaulieu M Reyes-Moreno C Guillemette C Bailey JL. Boar sperm storage capacity of BTS and androhep plus: viability, motility, capacitation, and tyrosine phosphorylation. Theriogenology. (2004) 62:874–86. 10.1016/j.theriogenology.2003.12.006 15251239

[37] Mortimer ST Mortimer D. Kinematics of human spermatozoa incubated under capacitating conditions. J Androl. (1990) 11:195–203. 10.1002/J.1939-4640.1990.TB03228.X 2384341

[38] Carling D. AMPK signalling in health and disease. Curr Opin Cell Biol. (2017) 45:31–7. 10.1016/j.ceb.2017.01.005 28232179

[39] Hardie DG Schaffer BE Brunet A. AMPK: an energy-sensing pathway with multiple inputs and outputs. Trends Cell Biol. (2016) 26:190–201. 10.1016/j.tcb.2015.10.013 26616193 PMC5881568

[40] Martin-Hidalgo D de Llera AH Calle-Guisado V Gonzalez-Fernandez L Garcia-Marin L Bragado MJ. AMPK function in mammalian spermatozoa *Int J Mol Sci*. (2018) 19:3293. 10.3390/ijms19113293 30360525 PMC6275045

[41] Hurtado de Llera A Martin-Hidalgo D Gil MC Garcia-Marin LJ Bragado MJ. AMP-activated kinase AMPK is expressed in boar spermatozoa and regulates motility. PLoS ONE. (2012) 7:e38840. 10.1371/journal.pone.0038840 22719961 PMC3375287

[42] Hurtado De Llera A Martin-Hidalgo D Rodriguez-Gil JE Gil MC Garcia-Marin LJ Bragado MJ. AMP-activated kinase, AMPK, is involved in the maintenance of plasma membrane organization in boar spermatozoa. Biochimica et Biophysica Acta (BBA) Biomembranes. (2013) 1828:2143–51. 10.1016/j.bbamem.2013.05.026 23747367

[43] Hurtado de Llera A Martin-Hidalgo D Gil MC Garcia-Marin LJ Bragado MJ. AMPK up-activation reduces motility and regulates other functions of boar spermatozoa. Mol Hum Reprod. (2015) 21:31–45. 10.1093/molehr/gau091 25281642

[44] Ronnebaum SM Patterson C Schisler JC. Minireview: hey U(PS): metabolic and proteolytic homeostasis linked *via* ampk and the ubiquitin proteasome system. Mol Endocrinol. (2014) 28:1602–15. 10.1210/me.2014-1180 25099013 PMC4179629

[45] Brown PR Miki K Harper DB Eddy EM. A-kinase anchoring protein 4 binding proteins in the fibrous sheath of the sperm flagellum. Biol Reprod. (2003) 68:2241–8. 10.1095/biolreprod.102.013466 12606363

[46] Carrera A Moos J Ning XP Gerton GL Tesarik J Kopf GS. Regulation of protein tyrosine phosphorylation in human sperm by a calcium/calmodulin-dependent mechanism: identification of a kinase anchor proteins as major substrates for tyrosine phosphorylation. Dev Biol. (1996) 180:284–96. 10.1006/dbio.1996.0301 8948591

[47] Eddy EM O'Brien DA. The spermatozoon. In:Kenobi E Neill JD, editors. The Physiology of Reproduction. New York, NY: Raven Press (1994). p. 29–77.

[48] Carnegie GK Means CK Scott JD. A-kinase anchoring proteins: from protein complexes to physiology and disease. IUBMB Life. (2009) 61:394–406. 10.1002/iub.168 19319965 PMC2682206

[49] Hillman P Ickowicz D Vizel R Breitbart H. Dissociation between AKAP3 and PKARII promotes AKAP3 degradation in sperm capacitation. PLoS ONE. (2013) 8:e68873. 10.1371/journal.pone.0068873 23894359 PMC3720880

[50] Lin RY Moss SB Rubin CS. Characterization of S-AKAP84, a novel developmentally regulated A kinase anchor protein of male germ cells. J Biol Chem. (1995) 270:27804–11. 10.1074/jbc.270.46.27804 7499250

[51] Vijayaraghavan S Liberty GA Mohan J Winfrey VP Olson GE Carr DW. Isolation and molecular characterization of AKAP110, a novel, sperm-specific protein kinase A-anchoring protein. Mol Endocrinol. (1999) 13:705–17. 10.1210/mend.13.5.0278 10319321

[52] Moss SB Turner RMO Burkert KL Butt HVS Gerton GL. Conservation and function of a bovine sperm a-kinase anchor protein homologous to mouse AKAP82. Biol Reprod. (1999) 61:335–42. 10.1095/biolreprod61.2.335 10411509

[53] Reinton N Collas P Haugen TB Skalhegg BS Hansson V Jahnsen T. Localization of a novel human a-kinase-anchoring protein, hAKAP220, during spermatogenesis. Dev Biol. (2000) 223:194–204. 10.1006/dbio.2000.9725 10864471

[54] Vizel R Hillman P Ickowicz D Breitbart H. AKAP3 degradation in sperm capacitation is regulated by its tyrosine phosphorylation. Biochimica et Biophysica Acta (BBA) General Subjects. (2015) 1850:1912–20. 10.1016/j.bbagen.2015.06.005 26093290

[55] Zapata-Carmona H Baron L Kong M Morales P. Protein kinase a (PRKA) activity is regulated by the proteasome at the onset of human sperm capacitation. Cells. (2021) 10:3501. 10.3390/cells10123501 34944009 PMC8700002

[56] Mahony MC Gwathmey T. Protein tyrosine phosphorylation during hyperactivated motility of cynomolgus monkey (*Macaca fascicularis*) spermatozoa. Biol Reprod. (1999) 60:1239–43. 10.1095/biolreprod60.5.1239 10208990

[57] Si Y Okuno M. Role of tyrosine phosphorylation of flagellar proteins in hamster sperm hyperactivation. Biol Reprod. (1999) 61:240–6. 10.1095/biolreprod61.1.240 10377055

[58] Wang YY Sun PB Li K Gao T Zheng DW Wu FP. Protein kinases regulate hyperactivated motility of human sperm. Chin Med J. (2021) 134:2412–4. 10.1097/CM9.0000000000001551 34669633 PMC8654453

[59] Zapata-Carmona H Baron L Zuniga LM Díaz ES Kong M Drobnis EZ. The activation of the chymotrypsin-like activity of the proteasome is regulated by soluble adenyl cyclase/cAMP/protein kinase A pathway and required for human sperm capacitation. Mol Hum Reprod. (2019) 25:587–600. 10.1093/molehr/gaz037 31329238 PMC8055059

[60] Galantino-Homer HL Visconti PE Kopf GS. Regulation of protein tyrosine phosphorylation during bovine sperm capacitation by a cyclic adenosine 3',5'-monophosphate-dependent pathway. Biol Reprod. (1997) 56:707–19. 10.1095/biolreprod56.3.707 9047017

[61] Bajpai M Doncel GF. Involvement of tyrosine kinase and cAMP-dependent kinase cross-talk in the regulation of human sperm motility. Reproduction. (2003) 126:183–95. 10.1530/rep.0.1260183 12887275

[62] Luconi M Carloni V Marra F Ferruzzi P Forti G Baldi E. Increased phosphorylation of AKAP by inhibition of phosphatidylinositol 3-kinase enhances human sperm motility through tail recruitment of protein kinase A. J Cell Sci. (2004) 117:1235–46. 10.1242/jcs.00931 14996943

[63] Vijayaraghavan S Goueli SA Davey MP Carr DW. Protein kinase A-anchoring inhibitor peptides arrest mammalian sperm motility. J Biol Chem. (1997) 272:4747–52. 10.1074/jbc.272.8.4747 9030527

[64] Marquez B Suarez SS. Different signaling pathways in bovine sperm regulate capacitation and hyperactivation. Biol Reprod. (2004) 70:1626–33. 10.1095/biolreprod.103.026476 14766720

[65] McPartlin LA Suarez SS Czaya CA Hinrichs K Bedford-Guaus SJ. Hyperactivation of stallion sperm is required for successful *in vitro* fertilization of equine oocytes. Biol Reprod. (2009) 81:199–206. 10.1095/biolreprod.108.074880 19208544

[66] Zigo M Jonakova V Manaskova-Postlerova P Kerns K Sutovsky P. Ubiquitin-proteasome system participates in the de-aggregation of spermadhesins and DQH protein during boar sperm capacitation. Reproduction. (2019) 157:283–95. 10.1530/REP-18-0413 30620719

