# Supplementary material for: Modulatory effect of MG-132 proteasomal inhibition on boar sperm motility during in vitro capacitation
Source: Front Vet Sci. 2023 Mar 23;10:1116891. doi: 10.3389/fvets.2023.1116891 (PMC10077870; doi:10.3389/fvets.2023.1116891)
Supplement: Supplementary file 1 [file Data_Sheet_1.pdf]

Supplementary file for

Modulatory effect of MG-132 proteasomal inhibition on boar sperm motility during *in vitro* capacitation

Lenka Hackerova[1], Barbora Klusackova[1], Michal Zigo[2], Natalie Zelenkova[1], Katerina Havlikova[1], Romana Krejcirova[1], Marketa Sedmikova[1], Peter Sutovsky[2,3], Katerina Komrskova[4,5], Pavla Postlerova[1,4*] & Ondrej Simonik[4*]

[1]Department of Veterinary Sciences, Faculty of Agrobiology, Food, and Natural Resources, Czech University of Life Sciences Prague, Czech Republic

[2]Division of Animal Sciences, University of Missouri, Columbia, MO, USA

[3]Department of Obstetrics, Gynecology & Women's Health, University of Missouri, Columbia, MO, USA

[4]Laboratory of Reproductive Biology, Institute of Biotechnology of the Czech Academy of Sciences, BIOCEV, Vestec, Czech Republic

[5]Department of Zoology, Faculty of Science, Charles University, Prague, Czech Republic

***Correspondence:**
Ondrej Simonik
ondrej.simonik@ibt.cas.cz
Pavla Postlerova
postlerova@af.czu.cz

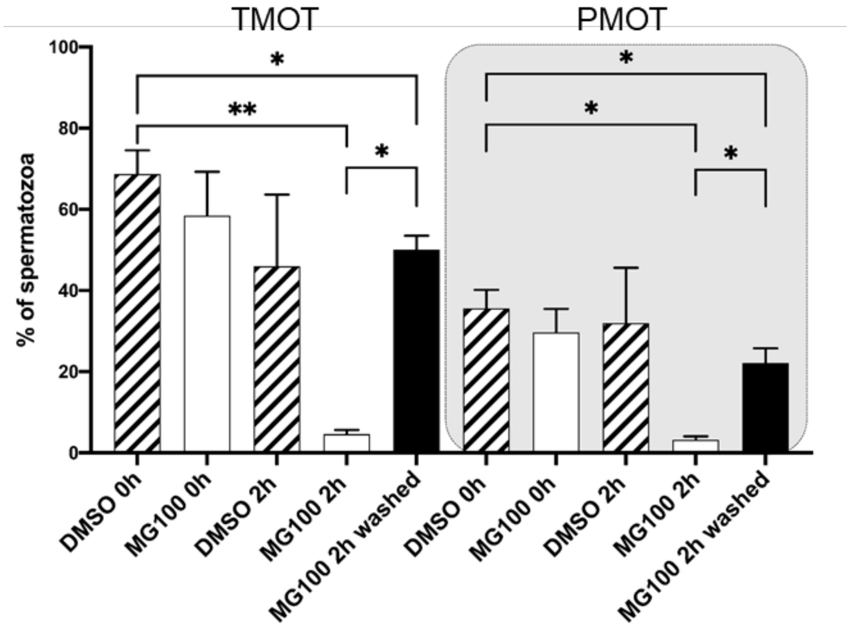

Supplementary Figure S1: Effect of inhibitor reversibility on total (TMOT) and progressive (PMOT) sperm motility. DMSO 0h, DMSO 2h – spermatozoa incubated without the inhibitor at 0 and 2 hours, respectively; MG100 0h, MG100 2h– spermatozoa incubated in CM with the MG-132 inhibitor at a concentration of 100 µM in time 0 and 2 hours, respectively; MG100 2h washed – spermatozoa incubated 2 hours in CM with the MG-132 inhibitor at a concentration of 100 µM, washed out from MG-132. Data are presented as mean ± SEM. Significance levels * $p \leq 0.05$, ** $p \leq 0.01$; n=4.

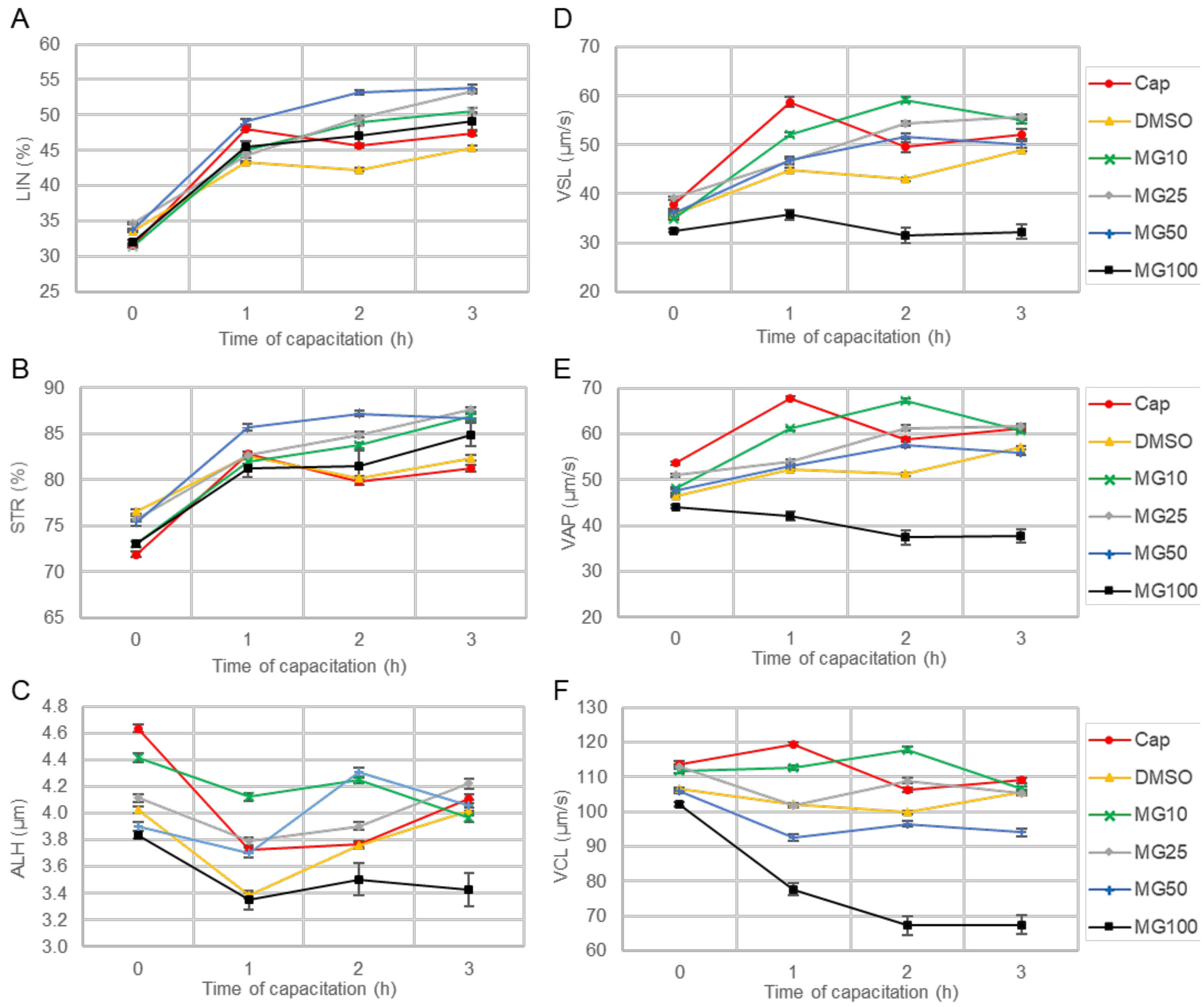

Supplementary Figure S2: Time course of kinematic parameters evaluation during sperm IVC under 26S proteasomal inhibition by MG-132 at various concentrations (10, 25, 50, and 100 μM) including capacitating (Cap) and vehicle (DMSO) controls. (**A**) LIN – linearity, (**B**) STR – straightness, (**C**) ALH – amplitude of lateral head displacement, (**D**) VSL – straight line velocity, (**E**) VAP – average path velocity, and (**F**) VCL – curvilinear line velocity. Data are presented as mean ± SEM; n=20.

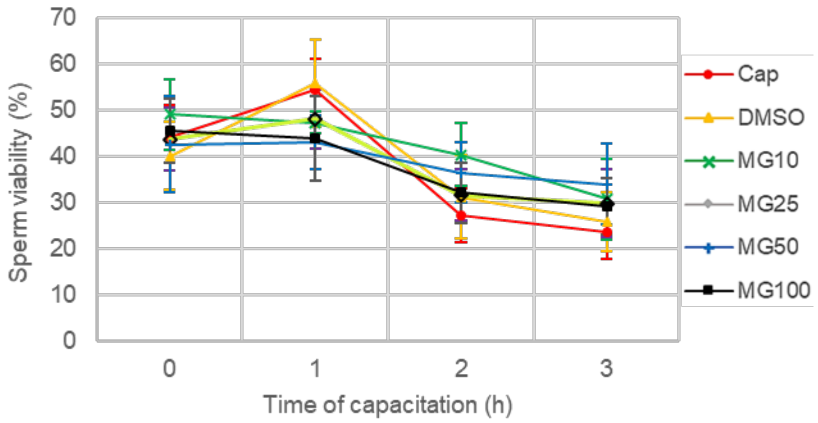

Supplementary Figure S3: Time course of sperm viability assessment during three hours of IVC under 26S proteasomal inhibition by MG-132 of various concentrations (10, 25, 50, and 100 µM). Control treatment groups comprised IVC spermatozoa incubated i) without both the inhibitor and vehicle (Cap) and ii) without the inhibitor (DMSO). Data are presented as mean ± SEM; n=20

Supplementary Video S1: Sperm movement after 2 hours of incubation with the inhibitor MG-132 in the capacitating medium at a concentration of 100 µM evaluated by CASA.

Supplementary Video S2: Sperm movement after 2 hours of incubation with the inhibitor MG-132 in capacitating media at a concentration of 100 µM and after a subsequent 15-minute equilibration in the capacitating medium without inhibitor evaluated by CASA system.
